# NICE’s early value assessment: an external assessment group’s commentary on the challenges and opportunities of NICE’s new life cycle approach to HealthTech

**DOI:** 10.1017/S026646232510055X

**Published:** 2025-10-23

**Authors:** Alan Lovell, Maxwell S. Barnish, Sophie Robinson, Caroline Farmer, Edward C.F. Wilson, Dawn Lee

**Affiliations:** 1 PenTAG, The Medical School, University of Exeter, Exeter, UK

**Keywords:** early value assessment (EVA), National Institute for Health and Care Excellence (NICE), evidence generation, HealthTech, Medtech

## Abstract

The National Institute for Health and Care Excellence (NICE) early value assessment (EVA) was launched in 2022 as a process to assess new technologies that have the potential to meet an unmet need or demand. The recommendations that result from the process are best viewed as a type of managed entry agreement – that is, time-limited and conditional on further evidence being generated. This commentary, from authors in PenTAG (an external assessment group involved in assessing medical technologies for NICE, based at the University of Exeter), explores the challenges that have arisen during 3 years of performing EVAs, offers some thoughts on EVA’s role in evidence generation, and their fit in NICE’s wider evidence landscape. The commentary identifies areas for potential improvement in terms of timelines, scoping and protocol development, searching, reviewing, and economic modeling. Many of the suggested changes are relatively minor tweaks to the process, or requests for clearer guidance or expectation management. We conclude that, with some changes to the EVA process and its accompanying guidance, the assessments could become more efficient. In summary, the EVA represents NICE’s life cycle approach in their HealthTech program, wherein evidence is collected along the life cycle to help monitor initial assumptions and recommendations made. The process is designed to continuously capture incremental innovation over the lifetime of a medical device. As such, EVAs reflect a small but important shift in how health technology assessment is practiced.

## Introduction

The National Institute for Health and Care Excellence (NICE) launched the early value assessment (EVA) process in 2022 to rapidly evaluate new technologies that could address unmet needs (1). MedTech, especially digital products, evolves quickly, and often emerges into the National Health Service (NHS) without clear usage guidance. EVA aims to address this by assessing clinical effectiveness and value for money in order to bring useful, cost-effective innovations to patients quickly (1). The potential of new technologies to tackle NHS challenges, like long waiting lists, is a frequent topic in media and policy discussions (2–4).

EVA topics are proposed by NHS England and via stakeholder engagement, and prioritized by NICE’s prioritization board process (1;5). They are typically low-risk interventions, like digital enhancements to existing rehabilitation or cognitive behavioral therapy services. Once a topic has been selected, an external assessment group (EAG) conducts rapid reviews of clinical and cost-effectiveness, identifies evidence gaps, and – normally – builds a *de novo* decision model (either a cost comparison or cost utility analysis). MedTech companies may submit evidence for consideration by the EAG. With the EAG report in hand, a NICE committee then reviews the evidence and decides for each technology to either (a) recommend for use in the NHS, (b) recommend for research only, or (c) not recommend for use. An evidence generation plan is typically prescribed alongside the recommendation, detailing the evidence that needs to be collected while the technology is used in the NHS, to support a full evaluation at a later date. EVA recommendations are, therefore, a type of managed entry agreement (6), that is, time-limited and conditional on further evidence being generated. There is no active funding mandate for technologies recommended by an EVA. However, this was the subject of a recent consultation (7) under NHS England’s 10 years plan (8).

Therefore, the EVA process addresses a key challenge in Health Technology Assessment (HTA): managing the health service’s use of rapidly evolving – but often evidence-lite – digital and MedTech products. This commentary, from PenTAG (an EAG based at the University of Exeter), reflects on lessons from their first 3 years of conducting EVAs, suggests process improvements, and considers EVA’s role in evidence generation and NICE’s broader evidence framework.

## EVA methods, challenges, and opportunities

At the time of writing, PenTAG has authored the EAG assessment report for five EVAs (Table 1). Here, we describe the challenges faced during the research and authoring process alongside suggestions for potential improvements. We have organized our description into five key areas of the process: timelines, scoping and protocol development, searching, reviewing, and economic modeling.

**Table 1.** EVAs performed by PenTAG

Title	HTA number	Technology assessed	Clinical articles included in the report	Economic studies included in the report	EAG de novo economic analysis	Recommendation
Guided self-help digital cognitive behavioral therapy for children and young people with mild-to-moderate symptoms of anxiety and low mood (1)	HTE 3 Published: 8 February 2023 Last updated: 5 September 2023	*N* = 4: 1 Internet-based intervention for ages 15–18 years with anxiety, low mood, or both 1 Internet-based parent-led and therapist-supported intervention for ages 5–12 years with anxiety 1 Internet-based intervention of cognitive therapy for ages 14–18 years with social anxiety 1 digital intervention in the form of a game (on Android and iOS) for ages 7–12 years with mild-to-moderate anxiety	6 completed studies 4 single-arm studies 1 RCT 1 redacted 6 ongoing studies 3 RCTs 1 single-arm study 2 redacted	*N* = 28: 8 EEs alongside RCT 7 systematic reviews 4 reviews 2 EE decision models 2 protocols 1 health state valuation 1 HTA assessment 1 meta-analysis 1 open pragmatic evaluation 1 editorial	Cost-utility analysis and value-of-information analysis	All four technologies can be used as an initial treatment option while evidence is being generated
Digitally enabled therapies for adults with depression (2)	HTE 8 Published: 16 May 2023 Last updated: 12 February 2024	*N* = 7: 3 online CBT for low mood/depression 3 online CBT for depression and anxiety 1 AI-supported CBT-based app for depression and anxiety	32 studies (46 papers) 14 RCTs 3 naturalistic studies 2 mixed methods 2 qualitative studies 2 IPD meta-analyses 2 pilot studies 2 meta-analyses 1 implementation study 1 RWE study 1 uncontrolled observational study 1 feasibility study 1 redacted	11 publications of direct relevance: 8 EEs alongside RCT 3 HTA assessments 21 publications of indirect relevance 5 systematic reviews 5 EEs alongside RCT 3 reviews 2 HTA assessments 1 EE decision model 1 health state valuation 1 meta-analysis 1 open pragmatic evaluation 1 protocol 1 editorial	Decision tree for the first 12 weeks, followed by a Markov model with year cycles, with a half-cycle correction applied	Two of the technologies can be used while further evidence is generated Three technologies should only be used as part of a research study. Other technologies are no longer available to the NHS
Virtual reality for treating agoraphobia and agoraphobic avoidance (3)	HTE 15 Published: 15 November 2023	*N* = 4: 1 a VR platform designed to be used by therapists to support treatment 1 VR app delivering cognitive therapy 1 VR app delivering CBT content and exposure exercises 1 a VR platform designed to be combined with face-to-face CBT	4 studies (9 papers) 2 single-arm studies 1 RCT 1 person-centered design process	1 publication of direct relevance: 1 EE alongside RCT	Decision analytic model (a two-state state-transition model (Markov model))	One of the technologies can be used while further evidence is generated Three technologies should only be used as part of a research study
Artificial intelligence auto-contouring for radiotherapy treatment planning (4)	HTE11 Published: 27 September 2023	*N* = 11: 8 standalone AI auto-contouring technologies 3 in-built AI auto-contouring technologies	15 prioritized studies 8 prospective studies 5 retrospective studies 1 prospective conference abstract 1 retrospective conference abstract	No publications of direct relevance	Cost-consequence analysis	Nine of the technologies can be used while further evidence is generated Two of the technologies were awaiting CE or UKCA mark approval, so they cannot be used yet
Digital technologies to support the delivery of pulmonary rehabilitation for adults with chronic obstructive pulmonary disease (5)	HTE18 Published: 30 April 2024	*N* = 7: 6 online platforms that support hybrid delivery of PR at home 1 Digital exercise program management software	9 prioritized studies 6 RCTs 1 observational study 1 conference abstract 1 redacted	No publications of direct relevance 3 publications of indirect relevance: 3 cost and resource use evaluations	Disaggregated cost-consequences analysis, complemented with exploratory cost-effectiveness analyses (decision analytic model)	One of the technologies can be used while further evidence is generated Five technologies should only be used as part of a research study One technology was awaiting appropriate regulatory approval

### Timelines

An EVA typically takes around 6 months from scoping to publication – assuming just one committee meeting is required (9). This includes 8 weeks for scoping, stakeholder identification, and specialist committee member recruitment; 9 weeks for external assessment (7 weeks to produce the draft report and 2 weeks to finalize), and 7 weeks for guidance production and public consultation. These streamlined timelines are designed for rapid assessment of limited evidence and preliminary economic modeling (9).

These timelines can, however, impact manufacturers’ capacity to participate in the assessment, such as providing comments on the draft protocol, submitting evidence in support of their technology, and responding to queries from the EAG. Timeline challenges may feel particularly acute to manufacturers that have not previously been through a NICE assessment. While the provision of information to NICE by manufacturers is optional, their input improves the quality of the assessment.

The timelines also necessitate the use of pragmatic approaches to the review and development of the economic model. These could include adapting existing published models, use of simplified model structures, focusing on shorter time horizons, use of targeted searches or expert opinion, use of threshold analysis, or a concentration on deterministic (rather than probabilistic) analysis. Whatever the approach, the model must focus on those areas that are of most importance for the determination of clinical and cost-effectiveness. It is, therefore, necessary that all stakeholders acknowledge the compromises required during a pragmatic appraisal.

*Suggestions for improvement:*

- Clearer guidance on when pragmatic steps are appropriate. For example, guidance on the maximum number of studies to be assessed, the appropriateness of evidence prioritization, or the production of only deterministic model estimates (or indeed whether modeling can/should be implemented). Such guidance should define what pragmatic means in respect of the ability to do an appraisal within available timelines, help decide when and what pragmatic steps are appropriate, and describe how to handle those cases where a pragmatic approach is not considered appropriate.
- Additional support for companies undergoing EVA for the first time. Ideally, this would include examples to follow for good practice.
- The creation of a formal step for presentation of the protocol to all stakeholders, with feedback provided during the presentation. This would allow discussion of any major technical objections to planned approaches prior to companies providing evidence, without introducing additional time into the assessment process.

### Scoping and protocol development

Scoping often raises challenges as there are typically multiple technologies available for a given indication (mean number of technologies across all final EVA scopes is currently 4.6, median 6.5, range 1–14), alongside multiple population subgroups and many outcomes of interest. While challenging to pin down, a broad scope is nevertheless useful for ensuring that assessments consider all the evidence useful for decision-making, including the way in which devices are implemented and used across a variety of real-world settings. Incorporating a wide variety of evidence types also increases the chance that evidence is identified for each technology.

The NICE team is responsible for the identification of technologies to be included in an assessment. This is a difficult task due to the complex nature of the MedTech market. On occasion, new treatments have been added to an EVA mid-process.

Once the NICE scope has been completed, the EAG produces a protocol that outlines the work that will be conducted. In general, once a protocol has been published, it is best practice to minimize further changes – to reduce the risk of bias and wasted resources. However, this is often not the case for EVAs, where changes to the protocols are commonplace due to the speed of scoping and protocol setting and the potential for changes to the scope mid-process (such as the addition of a new technology).

*Suggestions for improvement:*

- The separation of scoping and assessment timelines for EVAs. This would allow assessment to be scheduled around the reporting of key pieces of evidence, along with allowing time to decide on the most appropriate approach to assessment.
- Clearer guidance on how the NICE Information Specialist (IS) team and clinical experts describe challenges with the evidence during the scoping phase. If the evidence base is expected to be particularly large or complex, this would allow NICE to either adjust timeframes or edit the scope.

### Searching

EVA searches are designed and carried out quickly, so that the project can get underway speedily. The searches, however, also need to be suitably thorough to meet the needs of the broad scope typical of EVAs. This can be a tightrope to walk – information specialists are expected to produce structured searches that will not miss anything important, balanced with a need for speed and keeping the screening burden down. The searches need to provide a comprehensive and rigorous rapid review of the evidence base for both clinical and cost-effectiveness information.

This balance between thoroughness and speed is captured in the HealthTech program manual, which both acknowledges that a restricted number of databases may be searched, but also states that broad evidence mapping may be required because “articles may be published in less well-known journals, studies may not be well indexed or may only be presented as conference abstracts.” This broad evidence approach, with its focus on real-world evidence (RWE), takes time (e.g., randomised controlled trials [RCTs] can be searched for with greater precision than for RWE, using well-established search filters, plus there is an increasing ratio of observational studies being published per RCT (10)). Device names and companies may also change during the life of a product – impacting not only the search but the whole review – and there is often the expectation that the search includes the trawling of company websites for additional information. Even if device names are known, they are not always reported in titles and abstracts, and innovative technologies often do not yet have a common terminology.

In summary, searches in EVAs – while ostensibly limited and pragmatic in scope – need to be both broad and rapidly performed. Therefore, consultation with companies to confirm that the evidence base is suitably covered is often essential.

*Suggestions for improvement:*

- More tailored or structured Request for Information (RFI) forms to help companies unfamiliar with the process. For example, RFI forms could ask what other indications the device is used in and whether the technology has had alternative or previous versions and product names.
- The NICE IS team shares all scoping searches with the EAG before protocol development. This would help the EAG IS with the development of a search strategy.

### Reviewing

EAGs typically need to appraise more observational evidence in EVAs than is required in TAs on pharmaceuticals. Observational studies often require a more thorough and nuanced critique to assess relative merits and risk of bias compared with RCTs – hence, the selection and systematic appraisal of an EVA evidence base is generally more resource-intensive than in other appraisals.

Due to the broad evidence base, prioritization is often needed to focus on the most relevant studies – based on factors like randomization, sample size, and NHS relevance. EVA reports, therefore, aim to highlight high-quality, informative evidence, rather than covering everything available.

Finally, some of the information provided by companies may be confidential in nature. Any such confidential data need to be marked up as such in the assessment report. This is a shared responsibility, for companies are responsible for communicating the confidential information to NICE, NICE is then responsible for compiling a spreadsheet of confidential data, while the EAG is responsible for marking the confidential data in their report.

*Suggestions for improvement:*

- Companies to provide a table listing where confidential data can be found in the submission. This would facilitate the speedy and accurate marking of information in reports.
- Current NICE EVA guidance does not require assessment of risk of bias. We consider that the introduction of risk of bias could benefit EVAs if accompanied by clearer guidance for EAGs on when they can use rapid quality assessment tools. For example, the ROBINS-I quality assessment tool (11) for observational studies is resource-intensive to complete. Other tools, such as the SURE checklists, may be more appropriate for the objectives of an EVA (12).
- Companies to complete quality assessment for RWE when submitting an RFI. This would facilitate the inclusion of RWE into the evidence review and the decision model.
- A discussion of Artificial Intelligence (AI) is beyond the scope of this short commentary, but NICE’s position statement (13) makes clear that efficiencies are expected across the spectrum of HTA processes, while also acknowledging concerns about the appropriateness, transparency, and trustworthiness of AI.

### Economic modeling

The methods for EVA allow for either the construction of a *de novo* model or adaptation of an existing model (e.g., one produced by the NICE guidelines team). Guidelines also allow for a description/specification of a conceptual model where evidence is insufficient to perform analysis. It is rare for existing models to be available from industry, as MedTech companies have not been required to produce estimates of cost-effectiveness to access the UK market. Identification of existing models with the potential for adaptation must be conducted very early in the assessment timeframe. The suitability of an existing model for adaptation will be a function both of how well the model already fits the decision problem for the EVA and how user-friendly and transparent the existing model is. Between 1 September 2024 and 1 September 2025, nine EVAs were published or updated. Of these, two (14;15) reported the specification of a conceptual model rather than reporting results of an analysis, two (16;17) reported cost analyses, one (18) reported a narrative summary of costs and outcomes, and four (19–22) developed *de novo* or adapted existing models.

There is a particular challenge around the estimation of key parameters in EVAs. When a parameter is likely to have a large impact on the incremental cost-effectiveness ratio, structured expert elicitation methods are recommended to provide a more reliable estimate along with information on uncertainty (23–25). However, these exercises take time to run. They are unlikely to be feasible within a standard EVA timeframe unless only experts already recruited by NICE are included and training is initiated very early on in the assessment process. This requires an early decision on whether structured expert elicitation is going to be required – before the results of the evidence review are available.

Another challenge for EVA modeling – in fact, for all MedTech modeling when there is a life cycle focus to the assessment – is the need to consider costs associated with the set-up of new pathways. This may include costs for training, integration of any new information technology set-up with existing systems, and long-term maintenance. There is currently no guidance on how to quantify these types of costs in the NICE methods or Decision Support Unit documents. These types of costs are unlikely to be recoverable if use of the technology is stopped following the end of the EVA period. Additional cost may, in fact, be associated with taking technologies back out of use.

Full cost calculation is difficult within current timelines – company data must be carefully reviewed, and final prices are often unavailable to the EAG, as negotiation of the price is often conducted following the assessment. When the final price is not available, the maximum cost-effective price (economically justifiable price) can be calculated for an intervention (26).

Finally, the EVA timelines necessitate a single, simple model structure, likely focusing on the key value proposition of the technologies in question. Where the technologies have very different value propositions, each element will require a focus in the analysis, likely to be beyond the resource availability for an EVA. Therefore, it is important that all technologies considered within one EVA have a similar value proposition.

*Suggestions for improvement:*

- If an existing NICE model could be adapted, the people who constructed the model should brief the EAG before any model adaptation or decision on whether to reuse a guideline model.
- The CHEERS-AI checklist (27) to be used as a reporting standard for EVAs. Many of the considerations are equally applicable to devices and diagnostics that do not involve the use of AI.
- Clearer guidance for when the EAG can declare that an assessment of cost-effectiveness cannot be made due to a lack of evidence. This is likely to be appropriate where there is either no or highly limited evidence of clinical effect. For example, in PenTAG’s assessment of digital cognitive behavioural therapy (dCBT) for adults with depression, an assessment of cost-effectiveness was only possible for three out of seven interventions (19).
- Clearer guidance for when the EAG should seek input from clinical experts already recruited by NICE to inform model parameters. This is likely to be appropriate when information is required for a limited number of parameters and the cost of the technology is known. Experts will need to be trained and prepared.
- Where multiple technologies are assessed within the same EVA, they must have similar value propositions.
- Involvement from NHS England and clinical experts when calculating accurate costs. Together with the EAG, NHS England, and experts need to work up exactly what changes to pathways and practices will be necessary and the associated costs. Early involvement would inform modeling by clarifying clinical pathways and anticipating any barriers to adoption.

## EVAs and their role in evidence generation

A key step in producing an EVA involves reviewing evidence gaps and identifying data needed for future NICE reviews. This leads to the evidence generation that occurs off the back of the report and recommendation, which makes the EVA process so impactful. Evidence that needs to be collected is classified as “essential” or “supportive,” with guidance provided on potential study designs for generating it. The focus is on RWE collected within routine use rather than dedicated RCTs. Companies are responsible for ensuring data collection and analysis.

Companies must report their planned evidence generation activities within 6 months of NICE publishing an evidence generation plan. They then provide annual updates on data collection progress. Noncompliance may lead to guidance withdrawal by NICE. After the evidence generation period (most commonly 3 years), evidence is submitted for a decision on routine NHS adoption. Financial support was available through competitive funding facilitated by the Office for Life Sciences and the National Institute for Health and Care Research (NIHR) Invention for Innovation (i4i) program (28) – a single round of funding has supported seven research projects (Table 2) (29). Further rounds of funding have not yet been confirmed.

**Table 2.** Research projects funded by i4i subsequent to an EVA recommendation

Title	Institution	Relevant EVA(s)	Brief description
Internet-enabled cognitive behavioral therapy (CBT) for adults with depression or anxiety disorders	University of Sheffield	HTE8 HTE16	To compare internet-delivered CBT (iCBT) to individual or group CBT, to see how well iCBT works in the NHS, and whether it offers value for money. The research will also look at how well iCBT works across different groups of people
Evaluating digitally enabled cognitive therapies for post-traumatic stress disorder (PTSD) and social anxiety disorder	University of Oxford	HTE3	To compare internet-delivered cognitive therapies for social anxiety disorder and PTSD to usual NHS treatment, to see if it is possible to offer patients a wider choice of treatments in the future
Long-term impact of using the digital tool Gro Health W8Buddy	University of Warwick	HTE14	To compare outcomes for people using the Gro Health W8Buddy system to those receiving standard NHS care from weight management services
Clinical and cost-effectiveness of digital technology for low back pain	University of the West of England, Bristol	HTE16	To assess if the getUBetter app helps to improve pain and patients’ ability to engage with daily activities, and whether it offers good value for money for the NHS
Integrating digital innovation in weight management	Guy’s and St Thomas’ NHS Foundation Trust and Health Innovation Network South London, and King’s College London	HTE14	To test a digital weight management program called Roczen with patients in SE London. The researchers will look at various ways to use the program, including offering it to patients on waiting lists for specialist obesity care, and to patients who are leaving specialist care
Gamechange VR for patients with severe mental health difficulties	University of Oxford	HTE15	To test gameChange, a virtual reality (VR) treatment for people with psychosis, to see how well it works, and whether it is inclusive and affordable
Pharmacogenetics to avoid loss of hearing (PALOH-UK)	Manchester University NHS Foundation Trust	HTE6	To investigate the wider use of a genetic test that can determine which babies can safely receive an antibiotic called gentamicin, which can sometimes cause hearing loss. The research will be conducted across 14 neonatal units across the UK

**Figure 1.**
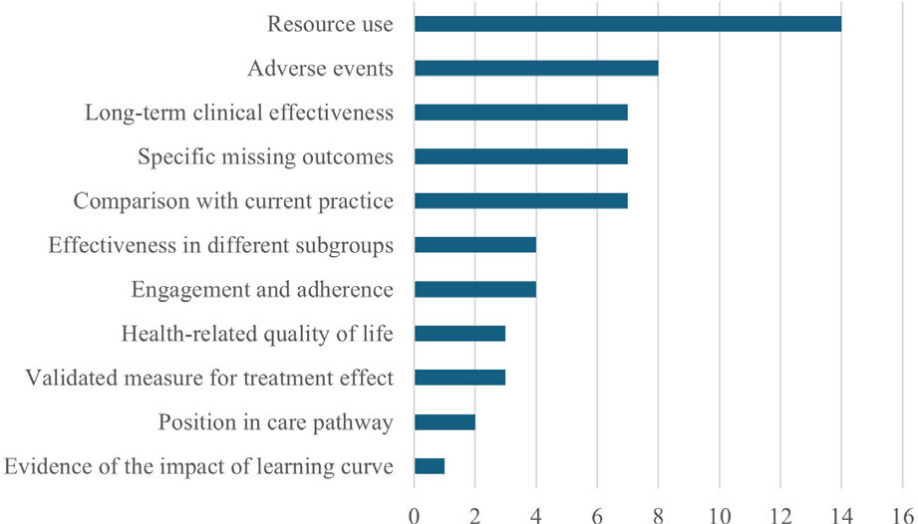
Areas flagged as “essential” for data collection in EVA evidence generation plans.

**Figure 2.**
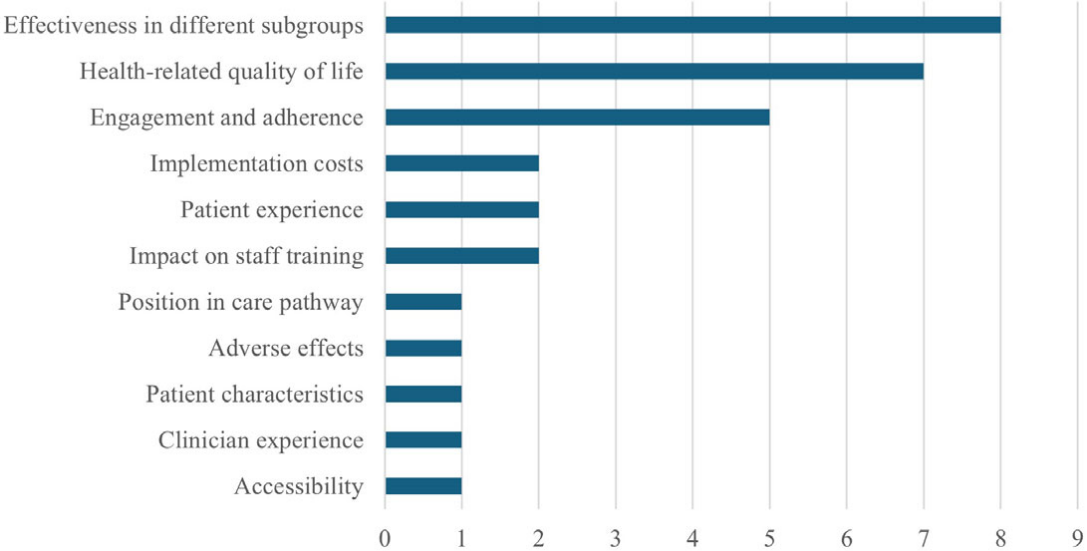
Areas flagged as “supportive” for data collection in EVA evidence generation plans.

As of writing (2 May 2025), NICE has published twenty-one EVAs, with nineteen recommended for evidence generation and seventeen with evidence generation plans available. Of these, eleven were expected to provide evidence within 3 years, three within 2 years, one within 4 years, and two did not state a time period. Key research areas for “essential” evidence included resource use, adverse events, and treatment impact on either long-term clinical effectiveness, outcomes unavailable in the original assessment, or in comparison with current practice (Figure 1). Of the EVAs, where areas for “supportive” evidence collection to aid future decision-making were included (Figure 2), the key areas of interest were effectiveness in different subgroups, health-related quality of life, and engagement and adherence.

**Figure 3.**
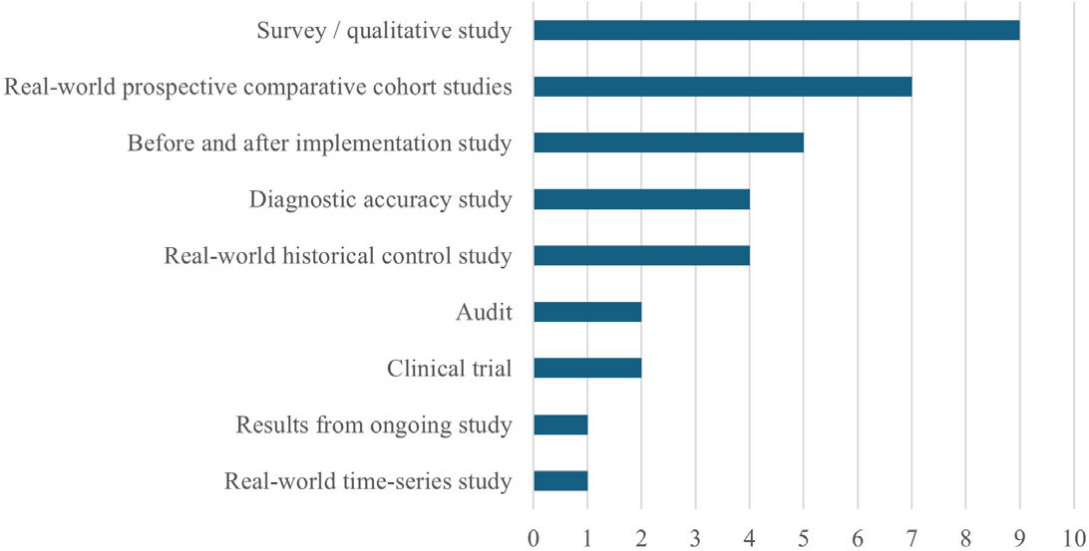
Type of data collection specified in EVA evidence generation plans.

Compared to data collection agreements for pharmaceuticals, EVAs generally have a greater level of detail in terms of outcomes that should be collected and the setting in which data collection should occur. However, there are no agreed quality standards in EVA agreements, and no clear consequences to suboptimal data collection (apart from an increased risk of a negative recommendation). We are, therefore, concerned that we may see a repeat of pharmaceutical-sector issues, such as widespread noncompliance with agreed data requests, failure to address key uncertainties within data collection, and highly variable quality of evidence collected (30;31). This is a particular issue for EVAs as reporting standards for medical devices have generally been low, with persistent gaps in the type and quality of evidence despite recent positive trends. (32) Planned data collection in evidence generation plans mostly relies on RWE (Figure 3). Challenges are compounded by limited NHS datasets for reassessment, and funding being restricted to a few technologies.

There is a role for value of information analyses (VoI) to guide the evidence generation following an EVA, but NICE does not mandate this. VoI assesses parameter uncertainty and quantifies the potential value of further research in terms of how much it is predicted to reduce uncertainty and hence the probability of a “wrong” adoption decision (33;34). Barriers to the adoption of VoI include a lack of expertise and time constraints, although knowledge of the technique is growing, and statistical approximations are available (35). Probabilistic analysis is required to generate inputs to calculate the VoI, which can itself be computationally expensive. However, the use of such analyses is uniformly recommended for early models (36), and all but the simplest models require probabilistic analysis to generate unbiased outputs (37).

Finally, it is desirable that materials produced for EVA can be reused in the reassessment. Systematic reviews can generally be updated due to the detailed reporting (including line-by-line search terms for all databases searched), but meta-analyses are harder to update as they often require effort to acquire the code used. While NICE receives economic models produced by EAGs, their simplified structure may require significant modifications for reassessment, limiting time savings. EVA models are developed to establish whether there is a *prima facie* case for adoption rather than to generate a definitive estimate of cost-effectiveness. There is a high chance that substantive structural changes would be necessary to accommodate new data and relevant elements to the decision, making adaptation rather than *de novo* analysis unlikely to save time.

*Suggestions for improvement:*

- Quality standards and specific agreed timelines are required for EVA data collection. Without these, companies will not face any clear consequences for suboptimal data collection.
- Where viable, consider using VoI analysis (33) to inform whether evidence generation is likely to yield a positive net benefit. This can be adapted to explicitly incorporate the cost of reversing decisions (e.g., where pathways need to be adapted) (34).
- Materials produced for EVA should be made available in a manner designed for reuse. For example, systematic literature reviews and any meta-analysis can easily be updated, and economic models can be adapted where this is considered appropriate and useful.

## Where might EVAs best fit in NICE’s evidence landscape?

The objective of an EVA is to identify interventions with a “promising signal” of cost-effectiveness early and promote the adoption while further evidence is generated. The adoption decision is then revisited once evidence is available. This contrasts with the traditional approach of waiting for “definitive” evidence. Both approaches have risks and benefits. The traditional approach places financial risk on the innovator and avoids NHS opportunity costs if the innovation proves non-cost-effective. However, if the innovation is indeed cost-effective, patients forego the opportunity to benefit during the evidence generation period. Conversely, the EVA approach transfers the financial and opportunity cost risk to NHS patients, especially when NHS funds, such as the NIHR i4i program, support evidence generation (NIHR funding is top-sliced from the NHS budget). There is also an opportunity cost risk from adopting a non-cost-effective intervention. In both cases, these costs manifest in terms of foregone health gain to other patients as resources are reallocated to new interventions, delaying or deferring other patients’ care. It is a judgment call as to whether the benefits of early adoption outweigh the risks/costs, which ultimately is the task of the appraisal committee.

NICE has to strike a balance between light-touch, low-cost, speedy exploratory assessments versus slower, more comprehensive assessments. It is helpful to draw a comparison between EVAs and managed access for pharmaceuticals. Both processes may result in reimbursement during evidence generation, but the standard of evidence and the assessment timelines are very different. This difference may be valid where topics are considered low risk, but low-risk/high-volume topics may carry similar aggregate risk to NHS patients as high-risk/low-volume topics such as cancer drugs appraised under the managed access processes.

In summary, EVAs provide top-level analyses to rule in or out technologies with clear cases, such as high-benefit/low-cost or questionable-benefit/high-cost interventions, aiming to detect whether there is a “signal” of cost-effectiveness. However, this approach comes with inherent limitations. For example, they cannot capture nuanced reasoning, exposing NICE to challenges from manufacturers claiming their evidence was overlooked or misunderstood. This carries both the risk of appeal against NICE committees as well as reputational risk for NICE, the EAGs, and the academic institutions hosting them. Mitigating these risks requires emphasis on the conditional nature of recommendations and recognition of the process’s limitations.

## Conclusions

EVAs have rapidly become part of NICE’s evidence landscape (38–42), offering early recommendations for new technologies and guiding research in key MedTech areas to fill in evidence gaps. This commentary outlines some challenges in producing EVA reports and suggests changes to improve efficiency.

EVA reviews and models are heuristic, aiming to identify a plausible case for cost-effectiveness and key drivers where evidence generation is needed. The process helps rule in strong candidates and “weed out” those that are unlikely to represent value for money, or for which the evidence base is still too immature. EVAs are not suitable for generating reliable estimates for borderline cases (although the incentive for industry is to price their products right at the buyer’s maximum willingness to pay, thus every carefully priced product will be a borderline case). Nevertheless, EVAs can signal acceptable NHS price ranges, encouraging more cost-effective innovation.

Timing an EVA is tricky – too early and no evidence exists; too late and multiple technologies may exceed scope and timelines. This dilemma is neatly summarized in “Buxton’s law”: “it is always too early for rigorous evaluation until, unfortunately, it is suddenly too late” (43). Each topic needs case-by-case consideration, and a qualitative survey of committee members to explore their views on the usefulness of modeling and other aspects of the EVA process would be a valuable next step.

EVA’s evidence generation component, followed by a full assessment to subsequently confirm routine access, aligns with NICE’s shift to a life cycle approach, which tracks innovation over time (44). This change in approach, therefore, marks a small but significant shift in how HTAs are done. However, it is still early days, and no EVA has yet completed its evidence collection, with all still in progress or planning. As timelines are typically around 3 years, we can expect to see the first EVA topics being reassessed in 2026.

## References

[1] National Institute for Health and Care Excellence (NICE). Early value assessment (EVA) for Medtech. London: NICE; 2024. Available from: https://www.nice.org.uk/about/what-we-do/eva-for-medtech.

[2] NHS England (NHSE). NHS AI expansion to help tackle missed appointments and improve waiting times. Leeds: NHSE; 2024. Available from: https://www.england.nhs.uk/2024/03/nhs-ai-expansion-to-help-tackle-missed-appointments-and-improve-waiting-times/.

[3] Department of Health and Social Care. Innovation and new technology to help reduce NHS waiting lists London: GOV.UK; 2021. Available from: https://www.gov.uk/government/news/innovation-and-new-technology-to-help-reduce-nhs-waiting-lists.

[4] Lovell T. Chancellor pledges more than £2bn for NHS tech and digital. London: Digital Health Intelligence Limited; 2024. Available from: https://www.digitalhealth.net/2024/10/chancellor-pledges-more-than-2bn-for-nhs-tech-and-digital/.

[5] National Institute for Health and Care Excellence (NICE). Our prioritisation decisions. London: NICE; 2025. Available from: https://www.nice.org.uk/about/what-we-do/prioritising-our-guidance-topics/our-prioritisation-decisions (accessed 28 Apr 2025).

[6] NICE Decision Support Unit. Managed entry agreements (MEA). Sheffield: University of Sheffield; 2016. Available from: https://www.sheffield.ac.uk/nice-dsu/methods-development/managed-entry-agreements.

[7] NHS England (NHSE). Building an integrated, rules-based medtech pathway [consultation]. Bristol: Citizen Space; 2024. Available from: https://www.engage.england.nhs.uk/specialised-commissioning/building-an-integrated-rules-based-medtech-pathway/.

[8] NHS England (NHSE). Fit for the future: 10 year health plan for England. London: NHSE; 2025. Available from: https://www.gov.uk/government/publications/10-year-health-plan-for-england-fit-for-the-future.

[9] National Institute for Health and Care Excellence (NICE). Early value assessment interim statement. Process and methods. 2022. London: NICE. Available from: https://www.nice.org.uk/process/pmg39/resources/early-value-assessment-interim-statement-pdf-72286784283589.

[10] Simoni L, Rizzoli S, Ciufici P, Ori A, Fiori G. Publishing observational studies vs randomized controlled trials – a PUBMED review. Value Health. 2019;22(3):S403–S940.

[11] Sterne JAC, Hernán MA, Reeves BC, et al. ROBINS-I: a tool for assessing risk of bias in non-randomised studies of interventions. BMJ. 2016;355:i4919. 27733354 10.1136/bmj.i4919 PMC5062054

[12] Specialist Unit for Review Evidence (SURE). Critical appraisal tools. Cardiff: SURE, Cardiff University; 2024. Available from: https://www.cardiff.ac.uk/specialist-unit-for-review-evidence/resources/critical-appraisal-checklists.

[13] National Institute for Health and Care Excellence (NICE). Use of AI in evidence generation: NICE position statement. London: NICE; 2025. Available from: https://www.nice.org.uk/about/what-we-do/our-research-work/use-of-ai-in-evidence-generation--nice-position-statement.

[14] National Institute for Health and Care Excellence (NICE). Artificial intelligence (AI) technologies for assessing and triaging skin lesions referred to the urgent suspected skin cancer pathway: early value assessment. Health technology evaluation. London: NICE; 2025. Available from: https://www.nice.org.uk/guidance/hte24.

[15] National Institute for Health and Care Excellence (NICE). Artificial intelligence-derived software to analyse chest X-rays for suspected lung cancer in primary care referrals: Early value assessment. Health technology evaluation. London: NICE; 2023. Available from: https://www.nice.org.uk/guidance/hte12.

[16] National Institute for Health and Care Excellence (NICE). Digital technologies to support self-management of COPD: early value assessment. Health technology evaluation HTE19. London: NICE; 2024. Available from: https://www.nice.org.uk/guidance/hte19.

[17] National Institute for health and care excellence (NICE). Robot-assisted surgery for soft tissue procedures: Early value assessment. Health technology evaluation HTE21. London: NICE; 2025. Available from: https://www.nice.org.uk/guidance/hte21.

[18] National Institute for Health and Care Excellence (NICE). Digital front door technologies to gather service user information for NHS talking therapies for anxiety and depression assessments: early value assessment. Health technology evaluation. London: NICE; 2025. Available from: https://www.nice.org.uk/guidance/hte30.

[19] National Institute for Health and Care Excellence (NICE). Digitally enabled therapies for adults with depression: Early value assessment. London: NICE; 2023. Available from: https://www.nice.org.uk/guidance/hte8.

[20] National Institute for Health and Care Excellence (NICE). Artificial intelligence technologies to help detect fractures on X-rays in urgent care: Early value assessment. London: NICE; 2025. Available from: https://www.nice.org.uk/guidance/hte20.

[21] National Institute for Health and Care Excellence (NICE). Digital therapy for chronic tic disorders and Tourette syndrome: early value assessment. Health technology evaluation. HTE25. London: NICE; 2025. Available from: https://www.nice.org.uk/guidance/hte25.

[22] National Institute for Health and Care Excellence (NICE). Robot-assisted surgery for orthopaedic procedures: Early value assessment. Health technology evaluation HTE22. London: NICE; 2025. Available from: https://www.nice.org.uk/guidance/hte22.

[23] National Institute for Health and Care Excellence (NICE). Interim methods and processes for late stage assessment (LSA) in HealthTech. In Development [GID-PMG10004]. London: NICE; 2024. Available from: https://www.nice.org.uk/guidance/indevelopment/gid-pmg10004.

[24] Horscroft J, Lee D, Jankovic D, Soares M, Bojke L. Structured expert elicitation for healthcare decision making: A practical guide. London: Lumanity; 2022. Available from: https://www.york.ac.uk/media/che/documents/Structured%20expert%20elicitation%20for%20healthcare%20decision%20making%20A%20practical%20guide.pdf.

[25] Centre for Health Economics. Structured expert elicitation course [Internet]. York: University of York; 2024. Available from: https://www.york.ac.uk/che/economic-evaluation/steer/.

[26] Economically Justifiable Price [Internet]. York: York Health Economics Consortium; 2016. Available from: http://www.yhec.co.uk/glossary/economically-justifiable-price/.

[27] Elvidge J, Hawksworth C, Avşar TS, et al. Consolidated health economic evaluation reporting standards for interventions that use artificial intelligence (CHEERS-AI). Value Health. 2024;27(9):1196–1205. 38795956 10.1016/j.jval.2024.05.006 PMC11343728

[28] National Institute for Health and Care Research (NIHR). i4i NIHR & OLS real world evidence guidance for applicants. London: NIHR; 2024. Available from: https://www.nihr.ac.uk/i4i-nihr-ols-real-world-evidence-guidance-applicants.

[29] National Institute for Health and Care Research (NIHR). NIHR and OLS invest £7.8m in new technology to benefit patients 2024. London: NIHR; 2024. Available from: https://www.nihr.ac.uk/news/nihr-and-ols-invest-ps78m-new-technology-benefit-patients.

[30] Pijeira Perez Y, Hughes DA. Evidence following conditional NICE technology appraisal recommendations: a critical analysis of methods, quality and risk of bias. PharmacoEconomics. 2024;42(12):1373–1394. 39249730 10.1007/s40273-024-01418-3 PMC11564307

[31] Trigg LA, Barnish MS, Hayward S, et al. An analysis of uncertainties and data collection agreements in the cancer drugs fund. Pharmacoecon Open. 2024;8(2):303–311. 38087151 10.1007/s41669-023-00460-9 PMC10883900

[32] Keane DF, Mathew C, Longley R, et al. Worldwide trends in the quality and breadth of clinical investigations of medical devices over the past decade: a scoping review and evidence map. Trials. 2025;26(1):113. 40158106 10.1186/s13063-025-08793-y PMC11955138

[33] Claxton K. Bayesian approaches to the value of information: implications for the regulation of new pharmaceuticals. Health Econ. 1999;8(3):269–274. 10348422 10.1002/(sici)1099-1050(199905)8:3<269::aid-hec425>3.0.co;2-d

[34] Eckermann S, Willan AR. Expected value of information and decision making in HTA. Health Econ. 2007;16(2):195–209. 16981193 10.1002/hec.1161

[35] Kunst N, Wilson ECF, Glynn D, et al. Computing the expected value of sample information efficiently: practical guidance and recommendations for four model-based methods. Value Health. 2020;23(6):734–742. 32540231 10.1016/j.jval.2020.02.010 PMC8183576

[36] Love-Koh J. How useful are early economic models? Comment on "problems and promises of health technologies: the role of early health economic modelling. Int J Health Policy Manag. 2020;9(5):215–217. 32563224 10.15171/ijhpm.2019.119 PMC7306112

[37] Wilson ECF. Methodological note: reporting deterministic versus probabilistic results of Markov, partitioned survival and other non-linear models. Appl Health Econ Health Policy. 2021;19(6):789–795. 34258732 10.1007/s40258-021-00664-2

[38] So D, Hearne E, Stapleton N, Chang-Douglass S. HTA186 accelerating access to medical technologies: an overview of the NICE early value assessment pilot project in the UK. Value Health. 2023;26(12):S355

[39] Morgan G, Bristow S. MT45 a review of the Recommendations & Evidence Gaps Reported across NIC’S early value assessments for Medtech. Value Health. 2024;27(12):S494

[40] Degraeve S, Degraeve S, Sharif H, Rinciog C. HTA149 from pilot to practice: insights from the NICE early value assessment for digital health technologies. Value Health. 2024;27(12):S382–S383.

[41] Carr D, Moran V. HTA17 NICE early value assessment: a new avenue for evidence generation and early market access in the digital therapeutics space in the United Kingdom. Value Health. 2024;27(12):S356

[42] Macaulay R, Carr D, Moran V. HTA5 an evaluation of the NICE early value assessment: what is the opportunity for digital therapeutics? Value Health. 2023;26(12):S320–S321.

[43] Buxton M. Problems in the economic appraisal of new health technology: the evaluation of heart transplants in the UK. In: Drummond MF (ed.). Economic appraisal of health Technology in the European Community. Oxford Medical Publications; 1987. p. 162–172

[44] National Institute for Health and Care Excellence (NICE). NICE HealthTech programme (consultation document). London: NICE; 2025. Available from: https://www.nice.org.uk/consultations/2857/1/introduction.

